# Comparative analysis of differentially secreted proteins in serum-free and serum-containing media by using BONCAT and pulsed SILAC

**DOI:** 10.1038/s41598-019-39650-z

**Published:** 2019-02-28

**Authors:** Jihye Shin, Jiheon Rhim, Yumi Kwon, Sun Young Choi, Sungho Shin, Chul-Won Ha, Cheolju Lee

**Affiliations:** 1 Center for Theragnosis, Korea Institute of Science and Technology, Seoul, 02792 Korea; 2 Department of Orthopedic Surgery, Samsung Medical Center, Sungkyunkwan University School of Medicine, Seoul, 06351 Korea; 3 Stem Cell & Regenerative Medicine Institute, Samsung Medical Center, Seoul, 06351 Korea; 4 Department of Life Science and Research Institute for Natural Sciences, Hanyang University, Seoul, 04763 Korea; 5 Department of Health Sciences and Technology, SAIHST, Sungkyunkwan University, Seoul, 06351 Korea; 6 KHU-KIST Department of Converging Science and Technology, Kyung Hee University, Seoul, 02447 Korea; 7 Division of Bio-Medical Science & Technology, KIST School, Korea University of Science and Technology, Seoul, 02792 Korea; 8 Present Address: Advanced Medical Research Center, Yokohama City University, Fukuura 3-9, 8 Kanazawa, Yokohama, 236-0004 Japan

## Abstract

Despite the increased interest in secretomes associated with paracrine/autocrine mechanisms, the majority of mass spectrometric cell secretome studies have been performed using serum-free medium (SFM). On the other hand, serum-containing medium (SCM) is not recommended very much because the secretome obtained with SCM is easily contaminated with fetal bovine serum (FBS) proteins. In this study, through the combination of bioorthogonal non-canonical amino acid tagging (BONCAT) and pulsed-SILAC (pSILAC), we analyzed differentially secreted proteins between SFM and SCM in a cancer-derived human cell, U87MG, and a mesenchymal stem cell derived from human Wharton’s jelly (hWJ-MSCs). In most cases, the bioinformatic tools predicted a protein to be truly secretory when the secretion level of the protein was more in SCM than in SFM. In the case of hWJ-MSCs, the amount of proteins secreted in SCM for 24 hours was larger than that of SFM (log_2_ fold change = 0.96), even considering different cell proliferation rates. hWJ-MSCs proteins secreted more in SCM included several positive markers of MSC paracrine factors implicated in angiogenesis, neurogenesis and osteogenesis, and upstream regulators of cell proliferation. Our study suggests the analysis of the secretome should be processed in SCM that promotes cell proliferation and secretion.

## Introduction

Cytokines, growth factors, and enzymes are secreted or released into culture medium or body fluids. The secretome that encompasses them all changes over time depending on the changes of environmental factors or disease state and can act as a reporter for the health state of a patient^1^. Therefore, it is important to understand the composition and dynamic changes of secretome during cell proliferation, development, and a certain pathological or environmental stimuli. They might also be a source of drug monitoring and disease diagnostic/prognostic biomarkers^2^.

The number of cell secretome studies has been increased for the past decade. However, many researchers have utilized serum-free media (SFM) to identify secreted proteins^3^. Cells growing under serum condition, usually 10% fetal bovine serum (FBS), are transferred to SFM and incubated for several hours before collection of the media for mass spectrometric (MS) analysis. Because the secreted proteins are mostly low abundant (as low as ng/mL) when compared to high abundant contaminating proteins derived from serum-containing culture media (~5 mg/mL), the FBS proteins often mask the low abundant secreted proteins, which makes it difficult to detect the secreted proteins by MS and interpret the profiling data^4^. Thus, serum starvation during cell culture has been used to collect secreted proteins without serum interference. Analysis of secretome in SFM reduces the complexity of the proteome leading to improved identification of secreted proteins. However, the cells undergoing serum starvation could disturb cell metabolism and proliferation and may increase the risk of cell cytolysis^5^. The washing step to reduce serum contaminants while changing the medium may also increase cell lysis. Thus, as a result of unintended biased experiments, contamination by cytoplasmic or other normally non-secretory proteins released following cell lysis and death, has often been disregarded in secretome analysis^6^.

To avoid distorting the analysis of secretome in SFM, a few research groups have attempted to analyze secretome in serum-containing media (SCM) in a way that reduces sample complexity^6,7^. pSILAC (pulsed stable isotope labeling with amino acid in cell culture) has been combined with BONCAT (bioorthogonal non-canonical amino acid tagging) which uses azidohomoalanine (AHA), an azide-bearing analogue of methionine, in order to enrich newly secreted proteins^8^. BONCAT exploits residue-specific incorporation of azide-containing label onto the newly synthesized proteins using the endogenous biosynthesis machinery without a need to modify the translation machinery by genetic engineering, and then copper (I)-catalyzed azide-alkyne cycloaddition (CuAAC) between the label-containing proteins and an alkyne-functionalized agarose resin^9^. pSILAC allows relative protein quantification by mass spectrometry^10^. Therefore, the combined BONCAT-pSILAC approach allows low abundant secreted proteins to be captured in the SCM, enabling their quantitation as well as identification^11^. Although BONCAT provides a mean to capture newly synthesized proteins, it is still very challenging to enrich such proteins secreted into SCM because fetal bovine serum proteins constitute the majority (it is estimated >99.99%) of total protein in SCM. Application of BONCAT to intracellular proteins has been performed occasionally for many years^8,9^. In contrast, only a few research groups (e.g. Eichelbaum *et al*.) have applied BONCAT-pSILAC to the analysis of secretome in SCM^11,12^. In this study, we adopted BONCAT-pSILAC and used a composite human-FBS database (HFDB) for the search of MS data^4^. The database was constructed by adding a list of experimentally validated FBS proteins (199 entries) to a reference human database. The reason for using such database was to strictly identify human cell line secreted proteins. That is, bovine serum proteins should not be misidentified as human proteins due to the tryptic peptides with homologous sequences between the two species. We analyzed differentially secreted proteins between SFM and SCM in U87MG glioblastoma cells and mesenchymal stem cells derived from human Wharton’s jelly (hWJ-MSCs). Most of the proteins secreted more in SCM than in SFM were predicted to be truly secretory by bioinformatics tools. hWJ-MSCs proteins secreted more in SCM included several positive markers of MSCs paracrine factors implicated in angiogenesis, neurogenesis and osteogenesis, and upstream regulators of cell proliferation. Our study suggests that analysis of the secretome in search of paracrine/autocrine factors needs to be processed in SCM.

## Results

### Comparison of the predicted secretion pathways of various secretomes

**Figure 1:**
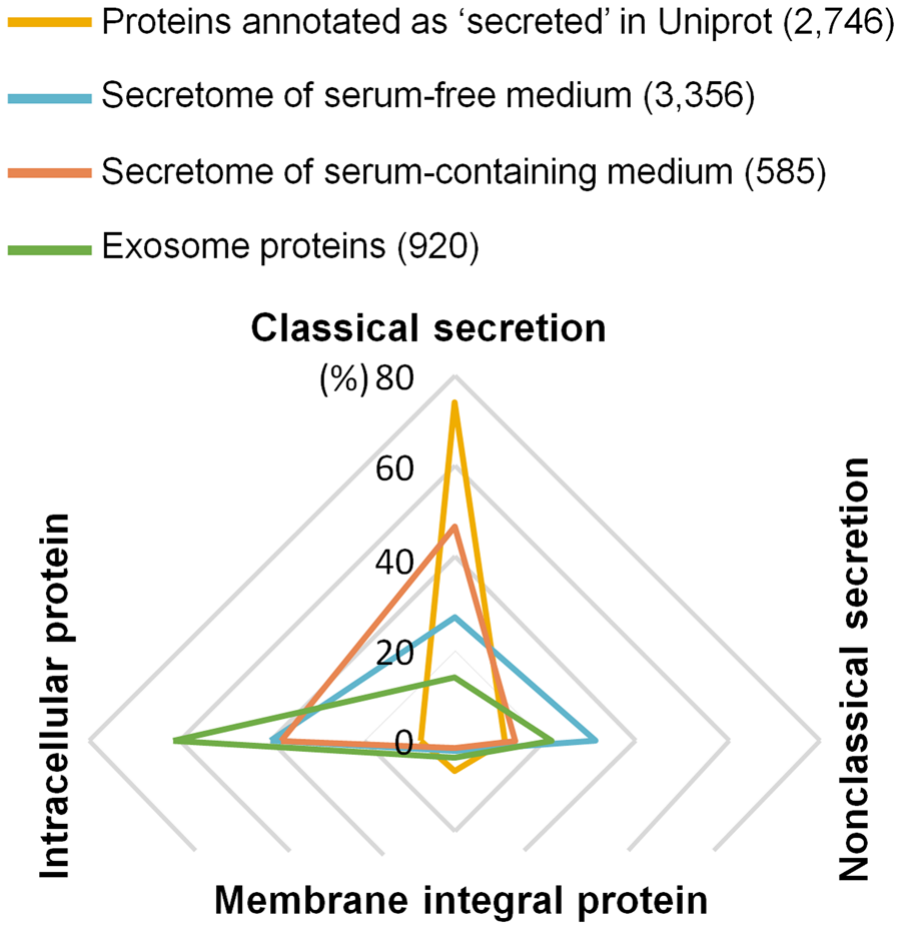
Comparison of the predicted secretion pathways of various secretomes. Four different secretomes were analyzed for their predicted secretion pathways by using three bioinformatics programs, SignalP, SecretomeP and TMHMM. Proteins in each secretome were classified into four categories based on the predicted secretion pathway (classical secretion, nonclassical secretion, integral to membrane, and intracellular). The four secretomes are (i) the proteins annotated as ‘secreted’ in the Uniprot protein database (2746 entries, yellow line), (ii) the secretome identified in the SFM of 12 cell lines (3356 proteins, blue line), (iii) the secretome analyzed after reducing the protein complexity of the SCM (585 proteins, orange line) and (iv) the exosome proteins reported by EVpedia to be cited in more than 100 papers (920 proteins, green line)

Although most of the cell secretome studies have widely utilized SFM, serum starvation is known to affect the amount of secreted proteins and their secretion pathways. To characterize the landscape of protein secretions at various conditions, we first compared the predicted secretion pathways of four different secretomes by using bioinformatics tools such as SignalP, SecretomeP and TMHMM (Fig. 1). The four secretomes were (i) the proteins annotated as ‘secreted’ in the Uniprot human protein database (2746 out of 20,316 reviewed entries), (ii) the secretome identified in the SFM of 12 cell lines in our previous studies (3356 proteins)^13–15^, (iii) the secretome analyzed after reducing the protein complexity of the SCM in the studies of other research groups (585 proteins)^6,16^ and (iv) the exosome proteins reported by EVpedia to be cited in more than 100 papers (920 proteins)^17^. The Uniprot secretome was predicted as secretory predominantly through classical secretion pathway (74.1%) by which proteins with signal peptides are secreted via ER and Golgi. Only a small portion was predicted to follow nonclassical secretion pathway (11.4%) and to be membrane integral proteins (6.7%). In contrast, the secretome of our previous studies identified in the SFM of 12 cell lines^13–15^ was predicted as 24.0% classical secretion, 34.9% nonclassical secretion, and 3.1% integral to the membrane. Exosome has been getting attention recently and our bioinformatics analysis showed that 61.4% of the exosome proteins were predicted as intracellular proteins, which indicates that exosome proteins are largely different from the secretome existing as soluble in the cell growth medium. The recently published papers concerning the studies of secretome in SCM^6,16^ revealed an increase of classical secretion than the secretome of SFM (Fig. 1). For this reason, we decided to compare the differentially secreted proteins between SCM and SFM conditions in a cancer-derived human cell and a mesenchymal stem cell by applying the combined BONCAT and pSILAC method, in which newly synthesized proteins were enriched and quantified after a specific treatment of serum starvation.

### BONCAT for analysis of the secretome

**Figure 2:**
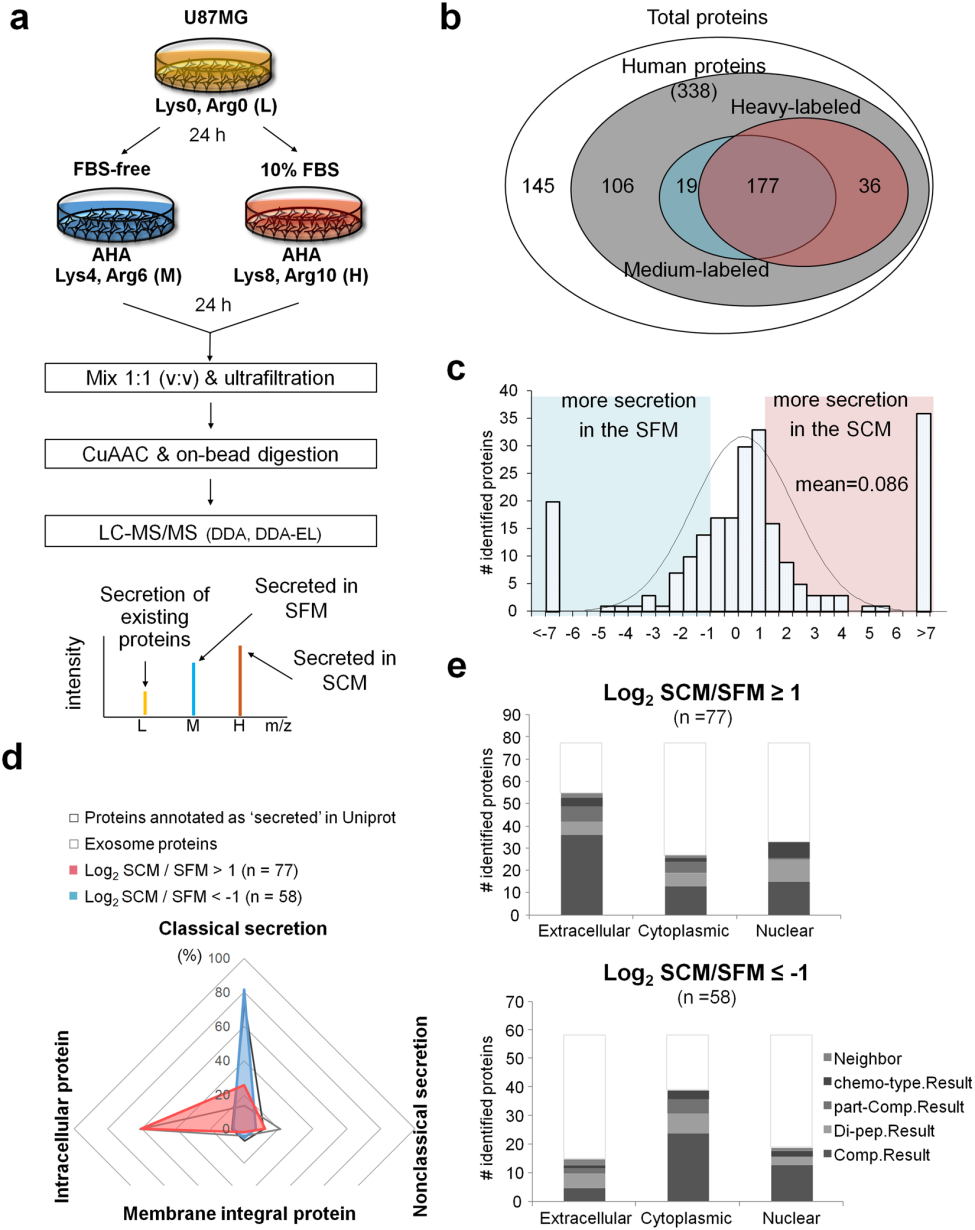
Analysis of U87MG secretome from serum-containing (SCM) and serum-free media (SFM). (**a**) Schematic workflow for quantitative analysis of U87MG secretome between SCM and SFM. DDA: data-dependent acquisition. DDA-EL: DDA with exclusion list. (**b**) The number of identified proteins in the secretome. (**c**) The distribution of differentially secreted proteins between SFM and SCM. The H/M ratios are log2-transformed after normalization by the difference in growth rate. (**d**,**e**) Secretion pathways and subcellular localization of the differentially secreted proteins. Secretion pathways were analyzed by using SignalP, SecretomeP, and TMHMM **(d)**, and subcellular localization by using Cello v2.5 (**e**).

The secretome of U87MG cells was pulse-labeled with AHA and SILAC amino acids for 24 h, in SFM and SCM separately. The two media were collected, mixed, and subjected to CuAAC enrichment (Fig. 2a). In preliminary experiments, we confirmed that U87MG showed no discernible differences in cell numbers and their morphology grown in the SCM supplemented with 1 mM methionine or 1 mM AHA (Supplementary Fig. S1). We also optimized the volume ratio between cell culture media and CuAAC resin to get as many proteins as possible: 20~30 mL of SCM for 100 µL of CuAAC agarose resin slurry (Supplementary Fig. S2). Although most FBS proteins in the medium would be washed out during enrichment process because they are not labeled with AHA, FBS protein contaminants derived from non-specific binding and incomplete washing are difficult to ignore considering the extremely low concentration of secretome (approximately 0.2~0.5 µg/mL)^4^. Therefore, we used the same composite database (HFDB) we had constructed previously for secretome analysis in SFM^4^. Database search using HFDB at 1% FDR generated 2.4-fold more PSMs than the search using human database only, and more than half of the total PSMs were matched to FBS proteins (Supplementary Fig. S3a). When we reanalyzed the data of Eichelbaum *et al*.^12^, in which they performed pSILAC-BONCAT to enrich secretome of a mouse cell, a similar phenomenon was observed (Supplementary Fig. S3b). This, as well as our result, suggests that a large amount of serum contamination still remained in the CuAAC-enriched sample. Therefore, we decided to use HFDB throughout the study to be more stringent in choosing truly secreted human proteins.

### Quantifying secreted proteins between serum-containing and serum-free media

A total of 483 proteins were identified in three replicate experiments using 30 mL of the mixed medium and 100 µL of resin per experiment. Among them, 338 proteins were annotated as human proteins. The enrichment process and the subsequent MS was reproducible, as judged by the identified and quantified proteins in each replicate experiment (Supplementary Fig. S4). The percentage of FBS contamination inferred by PSMs matched to bovine proteins was also similar between repeated experiments (Supplementary Fig. S5). Among the human proteins, 196 and 213 proteins were pulse-labeled with medium- and heavy-isotopes, respectively, and 177 proteins with both isotopes (Fig. 2b; Supplementary Table S1). In particular, 36 proteins were identified only in SCM as heavy-labeled, and of these, 34 (94.4%) proteins were predicted as true secretory proteins through the *in-silico* prediction programs such as SignalP, SecretomeP and TMHMM (see below). There were 19 proteins identified exclusively in SFM. In order to analyze differentially secreted proteins depending on the presence of serum in culture media, the H/M ratio of proteins were calculated and normalized by the difference in growth rate. U87MG cells had grown 1.55-fold more in the presence of serum during the 24-hr incubation time (Supplementary Fig. S6a). Although the level of protein secretion was greater in SCM than in SFM, the mean logarithm value of H/M ratio was 0.086 when normalized to account for faster cell growth in SCM (Fig. 2c). If two-fold cut-off was applied to the 135 pulse-labeled proteins, 77 proteins showed increased secretion in SCM, while 58 proteins showed decreased secretion. We classified the differentially secreted proteins according to their predicted secretion pathway and subcellular localization. The secretion pathway was predicted by using the three bioinformatics programs, SignalP, secretomeP and TMHMM. The subcellular localization was predicted using Cello v2.5. Among 77 proteins secreted more in SCM, 93.5% were predicted as true secretory proteins. The distribution of secretion pathways for the proteins secreted more in SCM was similar to that for the proteins annotated as ‘secreted’ in Uniprot. In contrast, only 39.7% of 58 proteins secreted more in SFM were predicted as true secretory proteins (Fig. 2d). When the pulse-labeled proteins were classified into three subcellular sites such as extracellular, cytoplasmic and nuclear regions, most of the SCM proteins were extracellular proteins, while the majority of SFM proteins were nuclear proteins (Fig. 2e). Consistent with this result, SCM proteins were mostly peptidase, transmembrane receptor, cytokine and growth factor, and the SFM proteins were mostly transcription and translation regulators (data not shown). Our results show that cellular protein secretory system is more active in the presence of serum and that abnormal release of intracellular proteins out of cells is blocked.

### Paracrine effect of stem cells in the presence of serum during co-culture

**Figure 3:**
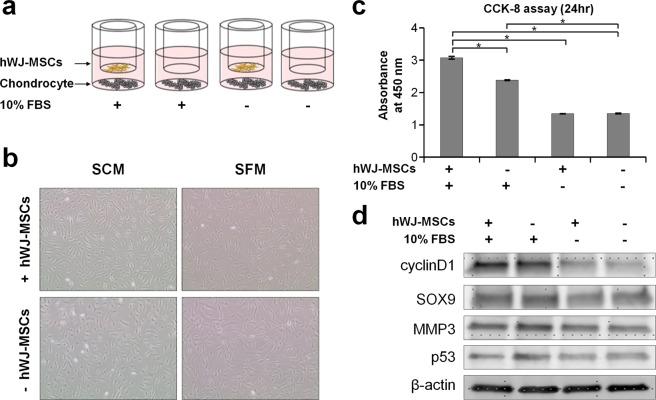
Effects of serum in media on proliferation of chondrocytes cocultured with hWJ-MSC. (**a**) Graphical representation of four different culture conditions. (**b**) Microscopic cell images of chondrocyte. **(c)** Cell proliferation assay performed by CCK-8 assay. Error bars are S.D. of triplicated experiments. (*p-value < 0.01). (**d**) Western blot images of cycinD1, SOX9, MMP3 and p53 proteins in chondrocytes. β-Actin was used as a control. Full-length images are shown in Supplementary Fig. S5a.

In a well-defined attempt to use MSCs for cartilage repair *in vitro*, traditional co-cultures that induce stem cells to release paracrine factors into chondrocytes require sera, which cannot be easily used in the proteomic analysis of secretome due to the masking effects of sera. However, secretome enrichment from SCM is expected to be applied to the analysis of specific cell changes upon the paracrine effect of stem cells even in the presence of serum. We first examined cellular changes of chondrocytes under co-culture with MSCs in the presence and absence of serum (Fig. 3a). The co-culture system was established by culturing hWJ-MSC on the upper layer and chondrocyte on the lower layer of a 6-well transwell plate for 24 h. Normal chondrocyte were cultured alone in SFM or SCM as a control. To determine if co-culture with hWJ-MSC in SCM induced proliferation of chondrocyte, we observed the chondrocytes under each condition by microscopy (Fig. 3b) and performed the CCK-8 assay, one of the most widely used markers of cell proliferation (Fig. 3c). The growth of chondrocyte increased clearly under the co-culture with hWJ-MSC in SCM, while, in SFM, chondrocyte did not grow well regardless of co-culture. In addition, western blot analysis showed that the expressions of cyclinD1, p53, SOX9 and MMP3 of chondrocyte co-cultured with MSCs in the SCM was higher than monoculture in SFM (Fig. 3d and Supplementary Fig. S7a). The expression of tumor suppressor p53 was reversed. These results suggest unknown paracrine factors of hWJ-MSC exist to mediate cell proliferation of chondrocytes in the SCM condition. We decided to apply our secretome enrichment technique to determine such paracrine factors.

### Secretome analysis of mesenchymal stem cells

**Figure 4:**
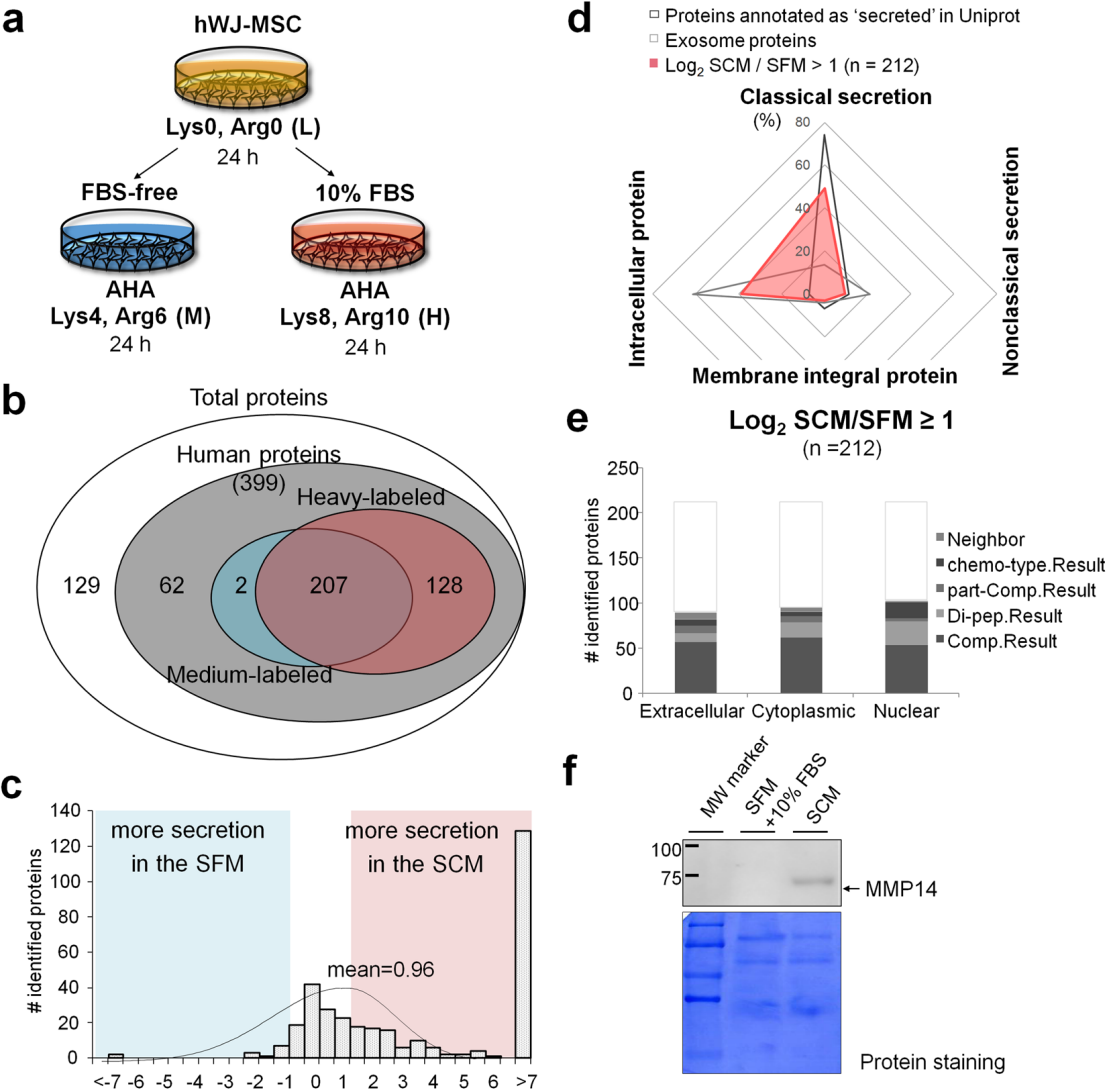
Analysis of hWJ-MSC secretome from serum-containing medium (SCM) and serum-free medium (SFM). (**a**) Schematic workflow. (**b**) The number of identified proteins in the secretome. (**c**) The distribution of differentially secreted proteins between SFM and SCM. The H/M ratios are log2-transformed after normalization by the difference in growth rate. (**d**,**e**) Secretion pathways and subcellular localization of the differentially secreted proteins. (**f**) MMP14 was measured by western blot analysis. Note that 10% FBS was added to SFM just before SDS-PAGE in order to view any background effect stemming from FBS itself. Equal loading was confirmed by Commassie staining of the membrane. Full-length western blot images are shown in Supplementary Fig. S5b.

Similar to the experimental setup for U87MG, growing hWJ-MSC was incubated with AHA and medium-isotope SILAC in SFM condition, and with AHA and heavy-isotope SILAC in SCM condition (Fig. 4a). Cell culture media were mixed by 1:1 ratio (v:v) and BONCAT enriched according to the workflow shown in Fig. 2a. We identified total 528 proteins in three replicate experiments (Supplementary Figs S4 and S5): 399 proteins were annotated as human proteins, among which 209 and 335 proteins showed medium-labeled and heavy-labeled peptides, respectively (Fig. 4b; Supplementary Table S2). Compared to U87MG experiment, the experiment with hWJ-MSC showed better reproducibility and lower FBS contamination (Supplementary Figs S4 and S5). In particular, 128 proteins showed heavy-labeled peptides only and 47.7% of them were predicted to be truly secretory by the bioinformatics tools, suggesting that they were synthesized and secreted after serum stimulation. On the contrary, there were only two proteins that showed medium-labeled peptides solely (Fig. 4b). We calculated H/M isotope ratio to examine which proteins were secreted more in SCM. As in the case of U87MG, the H/M ratio data were normalized by the difference of hWJ-MSC cell growth rate (1.78-fold; Supplementary Fig. S6b). The mean logarithm value of differentially secreted proteins was 0.96. That is the overall protein secretion was promoted 1.95 times as much in SCM (Fig. 4c). It was worth noting that the amount of secretion promoted in SCM of hWJ-MSC was more than those of glioblastoma cell U87MG. There were 212 proteins showing more than 2-fold increased secretion in SCM, of which 61.3% (130 proteins) were predicted as truly secretory proteins (Fig. 4d). Among the 212 proteins, 90 and 95 proteins were annotated as extracellular and cytoplasmic proteins, respectively (Fig. 4e).

In the secretome of hWJ-MSC, several surface proteins such as CD44, CDH2 and CD166, which were reported^18–21^ and well-reviewed^21,22^ previously as positive markers of mesenchymal stem cell, were secreted more in SCM (Table 1). Some other proteins implicated in angiogenesis, neurogenesis, osteogenesis and ECM homeostasis such as VEGFC, TGFB2, BDNF, TGFBI, THBS2 and MMP families^23–36^ were mostly increased in SCM compared to SFM. Increased secretion of one MMP protein, MMP14 was confirmed by western blot (Fig. 4f and Supplementary Fig. S7b). These results suggest that hWJ-MSC releases far greater amounts of paracrine factors that support proliferative activity in the presence of serum than without serum.

**Table 1:** Paracrine factors identified and quantified from hWJ-MSC secretome.

Paracrine factors	Secretion level log_2_(SCM/SFM)	References
**MSC surface marker**		
CDH2	0.94	^18^
CD166	1.91	^19^
CD44	1.23	^19, 20^
**Angiogenesis**, **neurogenesis**, **osteogenesis-related factor**		
VEGFC	↑^a^	^27, 30, 34^
TGFB2	↑	^24, 26, 27, 29, 34, 36^
BDNF	↑	^29, 33^
TGFBI	2.75	^24, 27, 29, 34, 36^
THBS2	1.30	^26^
**ECM homeostasis**		
MMP3	↑	^28, 29^
MMP10	↑	^23, 28^
TIMP3	↑	^28^
MMP14	2.39	^23, 28^

^a^Detected in SCM only.

### Bioinformatic pathway analysis of hWJ-MSC secretome

**Figure 5:**
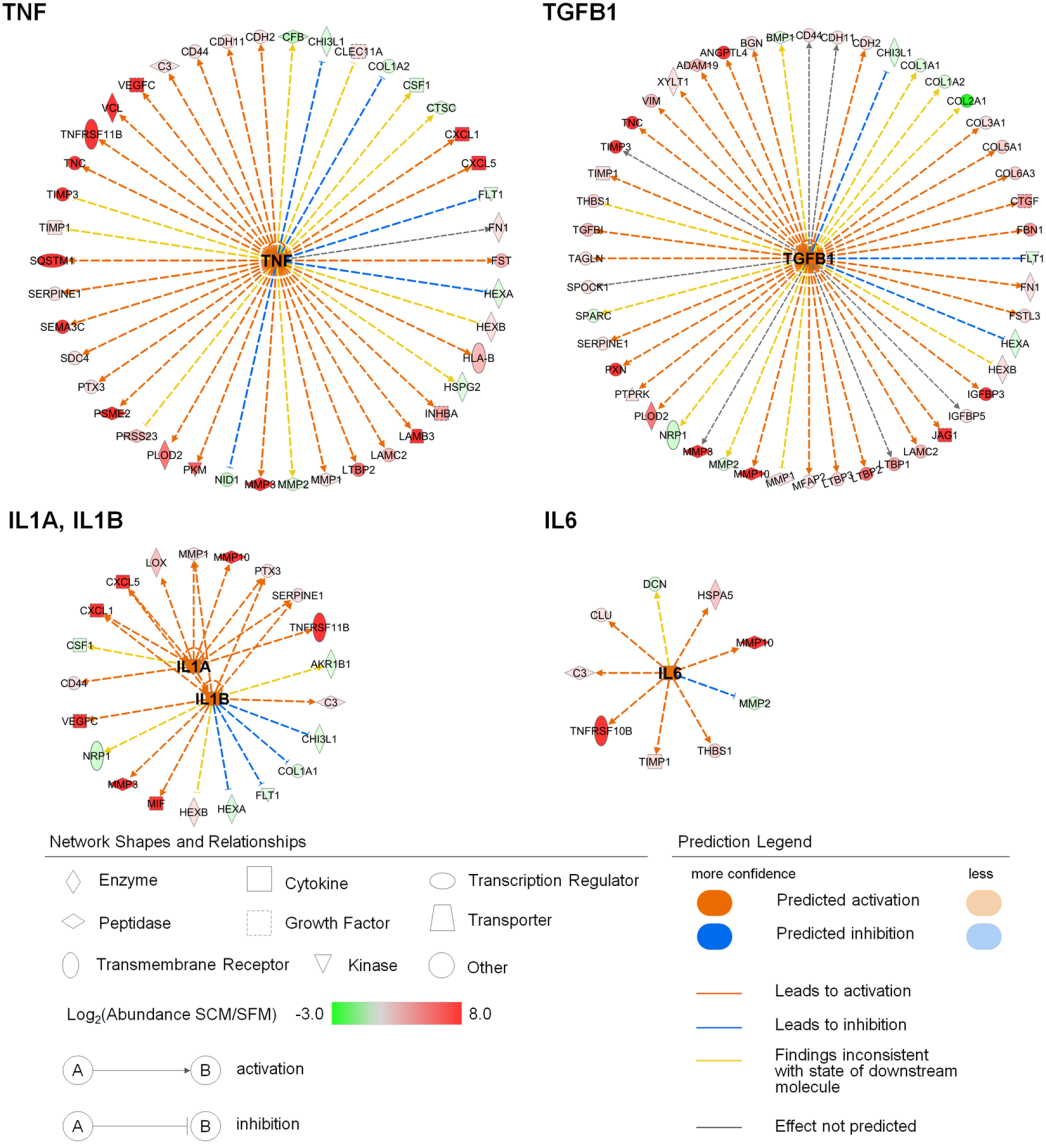
Upstream regulator analysis of hWJ-MSC secretome. Data illustrate the paracrine factors predicted as upstream regulators and their downstream targets in hWJ-MSC data sets. Activated upstream regulators (z-score ≥ 2) are highlighted in orange at the center of circular diagrams and the downstream targets are arranged along the circumference. Up-regulated and down-regulated proteins in SCM compared to SFM are highlighted in red and green, respectively. Orange and blue dashed lines with arrows indicate predicted direction of activation. Networks for IL1A and IL1B are merged in one network for simplicity.

We performed a bioinformatic analysis for the list of proteins identified in the secretome of hWJ-MSC using the Upstream Analysis module of Ingenuity Pathway Analysis (IPA). Upstream regulators are defined as molecules that can predict and explain the observed protein expressions in the dataset, based on the prior knowledge of expected effects between transcriptional regulators and their targets described in the IPA knowledge base. Moreover, the predicted activation state of the upstream regulator is made if the directions of observed fold-changes are mostly consistent with the activation state of the relevant regulator. Identified upstream regulators were predicted based on fold changes observed in SCM. Among these predicted upstream regulators that were connected to hWJ-MSC secretome, there were several paracrine factors which were well studied as commonly secreted from stem cells (Table 2)^37^. Five of these paracrine proteins (TNF, TGFB1, IL1A, IL1B and IL6) were computed out to be significantly activated (z-score ≥2). The predicted paracrine upstream regulators and their downstream targets in our secretome datasets are shown in Fig. 5. The activation states of these upstream regulators were mostly consistent with observations in the downstream dataset and the majority of the proteins secreted more or identified only in SCM were found in downstream of these upstream regulators. Therefore, we speculate that in the co-culture condition as depicted in Fig. 3, serum promotes secretion of paracrine factors from hWJ-MSC by activating such upstream regulators. The secreted factors may, in turn, exert a positive effect on the proliferation of chondrocytes.

**Table 2:** Upstream regulators of hWJ-MSC secretome known as stem cell paracrine factors.

Upstream Regulator^a^	Molecule Type	Activation z-score	*p*-value of overlap	Target molecules in the dataset
TNF	cytokine	3.333	3.44.E-15	42
TGFB1	growth factor	3.236	1.66.E-25	48
IL1B	cytokine	2.668	6.10.E-06	16
IL1A	cytokine	2.272	5.52.E-06	10
IL6	cytokine	2.045	1.60.E-03	9
COL18A1	other	0.213	9.75.E-06	9
HGF	growth factor	−0.285	2.34.E-07	13
MMP12	peptidase		1.36.E-11	13
MMP9	peptidase		2.28.E-03	3
TIMP3	other		2.59.E-03	2
TGFB2	growth factor		6.02.E-03	3

^a^Among the upstream regulators predicted to be linked to hWJ-MSC secretome, only those known as paracrine factors secreted commonly in stem cells are listed^37^.

## Discussion

Identification and quantification of secreted proteins have widely been accepted for secretome studies searching for diagnostic or drug monitoring markers that can be detected in serum or plasma^38^. Although hundreds of studies for secretome have been published, most of the current analysis of secreted proteins has been carried out in conditioned medium without FBS. Exclusion of FBS in secretome analysis is to minimize interference by background serum contaminant proteins. A previous study about cancer cell secretome from SFM reported that the viability and apoptosis of cell cultured in the SFM were little different until 24 h compared to those of cells grown in the SCM^39^. However, the uses of SFM have limited cell growth and thus may induce distorted results in the landscape of secretome. As shown in Fig. 1, 92.2% of the 2,746 human proteins annotated as ‘secreted’ in UniProt database (released Aug of 2017) were predicted as secretory proteins by the in-silico program e.g. SignalP, SecretomeP and TMHMM. Meanwhile, only about 45–55% of secretome were predicted as true secretory proteins in our previous studies and others on secretome in SFM^13–15,40,41^. In particular, about 30% of the identified proteins in the SFM were predicted to harbor signal peptides and thus be secreted through the classical secretion pathway. Other 30% of the proteins were predicted to follow the non-classical secretion pathway. These results of secretome in SFM may be due to low protein secretion and an increase of cytolysis during serum starvation. Another possibility is that serum starvation may stimulate unusual protein secretion which is not well characterized yet.

Several recent studies have attempted to analyze the secretome in the SCM to acquire the information of secreted proteins reflecting for real physiological state^6,16^. They analyzed the secretome in the SCM directly by using the MLEFF (Metabolic Labeling, protein Equalization, protein Fractionation, and Filter-aided sample preparation) strategy which combined SILAC, protein equalization by ProteoMiner, protein fractionation by molecular size, and filter-aided sample preparation (FASP) to reduce dynamic concentration range of proteome in SCM. Eichelbaum *et al*. pioneered a state-of-the-art technology by combining BONCAT and pSILAC in order for secretome analysis^11^. The method has been developed to identify and quantify newly synthesized proteins in the cells. The same research group also analyzed newly secreted proteins after macrophage activation^12^. Although the proteins without methionine may not be enriched by AHA labeling, such proteins constitute only about 1% of all entries in a human protein database. Also, about 5% of the human proteome possesses only a single, N-terminal methionine that may be removed by posttranslational modification. Therefore, this approach is applicable to about 94% of the mammalian proteomes^42^. In our study, we analyzed the quantitative difference of secretion between SFM and SCM, and the characteristics of differentially secreted proteins in order to find out the importance of secretome analysis in the SCM. Additionally, we used the human-FBS database in order to increase the true-positive identifications, because a plenty of serum contamination still remained in the enriched sample. Although the secretome profiles of the two cells, MSCs and cancer cells, were significantly different, the identified contaminant FBS proteins were similar, indicating that FBS contamination is almost unrelated to cell type (Supplementary Fig. S5c).

In a comparative analysis of the secretome between SFM and SCM, even considering cell growth during incubation time, the amount of secreted proteins in the SCM for 24 h was 1.87 times (hWJ-MSC) higher than those in the SFM (Fig. 4c). In addition, 90.7% (U87MG) and 60.6% (hWJ-MSC) of the proteins secreted more than two-fold higher in the SCM than in SFM were predicted to be truly secretory (Figs 2d and 4d). The result implies that FBS dependency of stem cells is higher than that of immortalized cancer cells. This is very likely given the nature of these two types of cells. It is also supported by various previous reports^43,44^. Therefore, this study suggests that the analysis of the secretome should be processed in the SCM because it resembles most of the molecular and cellular biology studies.

A recent paradigm for beneficial effects of stem cells has shifted to paracrine actions and not just differentiation^37^. Stem cell therapy represents a promising strategy in regenerative medicine. The secretome of hWJ-MSC, which contains a broad spectrum of cytokine, chemokines and growth factors implicated in angiogenesis, neurogenesis and osteogenesis, is being broadly studied in clinical trials. Hence, many researchers have demonstrated that MSC secreted factors are sufficient to demonstrate the MSC effects and provide the opportunity to exploit the potential therapeutics^30,37,45–48^. In this regard, our analysis of hWJ-MSC secretome in SCM was timely. Of note, several positive markers of MSCs, such as CD44, CDH2 and CD166^21,22,42^ were increased in SCM (Table 1). Many proteins implicated in angiogenesis, neurogenesis and osteogenesis were also increased in SCM. Furthermore, several paracrine factors well-reviewed in a paper^37^ were predicted as upstream regulators of the proteins secreted more in SCM than in SFM. The activation state of those upstream regulators was also consistent with expressional changes in response to serum. Hence, we demonstrated that the secretome analysis of MSC should be processed in presence of FBS to study actually promoted paracrine effects of MSC.

In this study, we have demonstrated that serum starvation has a marked effect on secretome composition and secretome analysis in SCM is important. The identification of proteins in responses to serum has demonstrated the potential of this approach to uncover paracrine factors and biomarkers aiming at tailored interventions in processes as a specific stimulation and response. Moreover, we expect this approach of BONCAT-pSILAC can be used not only to analyze the secretome in SCM but also to compare the differentially secreted proteins of multiple cells or upon a specific stimulation for discovering disease-specific or drug monitoring markers.

## Materials and Methods

### Cell culture and pulse-labeling with AHA and SILAC

U87MG was obtained from Korean Cell Line Bank (KCLB). hWJ-MSCs was kindly provided by Prof. Jong Wook Chang at Samsung Medical Center, Republic of Korea, and the cell cultivation was performed according to his previously published method^49^. The U87MG and hWJ-MSCs were cultured in DMEM (Gibco, Rockville, MD) supplemented with 10% FBS (Gibco, Rockville, MD), 1% penicillin and streptomycin (Gibco, Rockville, MD) at 37 °C in a humidified 95% air, 5% CO_2_ incubator. Cells were seeded 2.2 × 10^6^ cells in 100-mm culture dishes (Nunc, Naperville, IL) or 5 × 10^6^ cells in 150-mm culture dishes and incubated for 24 h. In the experiments of BONCAT optimization, the cultured cells were first depleted of methionine in methionine-free medium (Gibco) with 10% dialyzed FBS for 1 h and then incubation for 24 h in the same medium supplemented with 1 mM AHA (Invitrogen, Carlsbad, CA) and 10% dialyzed FBS. In the case of BONCAT-pSILAC experiments, cells were depleted of methionine, lysine, and arginine in a depletion medium (DMEM non-GMP formulation without methionine, arginine, and lysine; Gibco) with 10% dialyzed FBS for 1 h, and then incubated for 24 h in the same medium supplemented with 1 mM AHA and either 0.398 mM [^13^C_6_,^15^N_4_]L-arginine and 0.789 mM [^13^C_6_,^15^N_2_]L-lysine (Cambridge Isotope Laboratories, Inc.) as heavy-isotope with 10% dialyzed FBS or 0.398 mM [^13^C_6_]L-arginine and 0.789 mM [4,4,5,5-D_4_]L-lysine (Cambridge Isotope Laboratories, Inc.) as medium-isotope without FBS. After incubation, culture media were carefully collected. Floating cells and cellular debris were removed by centrifugation (400 × g, 10 min, 4 °C), followed by sterile filtration (pore size: 0.22 μm, Millipore, MA). Any media containing 5% or 10% FBS are referred as SCM, and any media without FBS as SFM.

### Filter-aided enrichment of newly synthesized proteins and on-bead digestion

Newly synthesized and secreted proteins were enriched from concentrated media using the Click-iT® Protein Enrichment Kit (Invitrogen C10416), employing the vendor’s protocol with slight modifications^11^. Typically, 100 µL of agarose resin slurry was used for concentrated SCM. To determine the appropriate volume of SCM for the CuAAC reaction, the enrichment experiments were performed at five different volume conditions (3, 10, 20, 30 and 60 mL) of 10% FBS-containing medium. The SCM was concentrated up to ~250 µL through ultrafiltration using ‘Amicon Ultra-15’ centrifugal filter devices (Millipore, MA), and was exchanged into a denaturation buffer containing 8 M urea and 100 mM Tris (pH 8.2) by repeating dilution-ultrafiltration twice. CuAAC reaction was carried out overnight at RT after mixing the sample with appropriate resin and solutions supplied by the vendor. The whole mixture adjusted to 0.5 ml with water was then transferred on to a 0.22-µm centrifugal filter unit (Millipore). All the water-soluble materials were removed by spinning the filter unit, leaving only the resin. After then, the resin with proteins attached was treated with 20 mM DTT in 0.5 ml of 1% SDS at 70 °C for 15 min, and then with 40 mM iodoacetamide in 0.5 ml of 1% SDS at RT for 30 min in the dark, and washed with 0.5 ml of 1% SDS, 0.5 ml of 8 M urea/100 mM Tris (pH 8.2) and 0.5 ml of 20% acetonitrile. Each washing step was repeated at least five times. The washed resin was resuspended in a buffer containing 100 mM Tris (pH 8.2), 2 mM CaCl_2_, and 10% acetonitrile, mixed with 0.5 µg trypsin and incubated for 16 h at 37 °C. The peptides of trypsin cleavage product were collected by centrifugation and the resin was washed with 0.5 mL of water. The two solutions were combined, acidified with 0.5% TFA, and analyzed by LC-MS/MS.

### Liquid chromatography and tandem mass spectrometry (LC-MS/MS)

LTQ-XL mass spectrometer (Thermo Scientific, San Jose, CA) was used during BONCAT optimization experiments. Peptide samples were reconstituted in 0.4% acetic acid and one-fifth of the sample was injected into a reversed-phase Magic C18aq column (15 cm × 75 μm, 200Å, 5U) on an Agilent 1200 HPLC system (Agilent Technology). The column was pre-equilibrated with 95% solvent A (0.1% formic acid in water) and 5% solvent B (0.1% formic acid in acetonitrile). The peptides were eluted at a flow rate of 0.4 μL/min with a linear gradient of 5–40% solvent B over 120 min. The ESI voltage was set to 1.9 kV, the capillary voltage to 30 V, and the temperature of the heated capillary to 250 °C. The MS survey was scanned from 300 to 2,000 m/z, followed by three data-dependent MS/MS scans with the following options: isolation width, 1.5 m/z; normalized collision energy, 25%; dynamic exclusion duration, 180 s. The optimization experiment was performed in duplicate, triplicate or quadruplicate.

Q Exactive mass spectrometer (Thermo Fisher Scientific) was used in the BONCAT-pSILAC experiments. One microgram of sample reconstituted in 0.4% acetic acid was injected into a reversed-phase C18 column (20 cm × 75 μm i.d., 3 μm, 120 Å, packed in-house; Dr. Maisch GmbH) on an Eksigent nanoLC-ultra 1D plus system at 95% solvent A and 5% solvent B. The peptides were eluted with a linear gradient from 5% to 40% solvent B over 200 min followed by 80% solvent B wash and 95% solvent A re-equilibration at a flow rate of 300 nL/min with a total run time of 230 min. Survey full-scan MS spectra (m/z 350–1800) were acquired at a resolution of 70000. Source ionization parameters were as follows: spray voltage, 2.5 kV; capillary temperature, 300 °C; and s-lens level, 44.0. The MS peak width at half height was <30 s. The MS/MS spectra of the 12 most intense ions from the MS1 scan with a charge state ≥2 were acquired with the following options: resolution, 17500; isolation width, 2.0 m/z; normalized collision energy, 27%; ion selection threshold, 4.00E ± 03 counts; and peptide match, ‘preferred’. The experiment was performed in triplicate. The mass spectrometry proteomics data have been deposited to the ProteomeXchange Consortium via the PRIDE partner repository with the dataset identifier PXD007529^50^.

### Analysis of mass spectrometric data

Raw data of LC-MS/MS were processed using Sequest HT in Proteome Discoverer 2.1.1.21 (Thermo Fisher Scientific Inc.). Human UniProtKB reference proteome database (released in January 2016; 20,218 entries) combined with a list of experimentally validated FBS proteins (199 entries) was used, unless otherwise indicated. In our previous publication^4^, the search result of secretome analysis data against such a composite human-FBS database (HFDB) was more reliable with fewer false-positive and false-negative identifications compared to using a human only database. Search parameters were two missed trypsin cleavage sites, cysteine carbamidomethylation as fixed modification, methionine oxidation and N-terminal protein acetylation as variable modifications. Peptide identification was performed with an allowed initial precursor mass tolerance up to 15 ppm and an allowed fragment mass deviation 0.05 Da. Peptide and protein results were filtered to 1% FDR. For the checking of AHA incorporation before enrichment experiment, AHA (−4.9863 Da) and l-2,4-diaminobutanoate (−30.9768 Da), a product of reduction of AHA, were specified as variable modifications for methionine.

In case of pSILAC data, Proteome Discoverer 2.2.0.388 was used with three search engines: Sequest HT, Mascot and MS Amanda. Each search engine was set to medium- and heavy-isotope of SILAC as variable modifications with precursor mass tolerance up to 15 ppm and an allowed fragment mass deviation of 0.05 Da. Only the proteins with at least one unique peptide supported by all three search engines were accepted into the final result list. The H/M ratios of peptides were calculated by dividing the intensities of heavy-isotope by the medium-isotope intensities and then transformed to log2 values. For missing value imputation, the smallest integer greater than the largest log2 peptide ratio was given: a value of −8 was given to the peptides whose heavy isotope was not observed; a value of 8 was given to the peptides whose medium isotope was not observed. Protein ratio was the geometric mean of all unique peptide ratios.

### Co-culture of chondrocyte and hWJ-MSCs

Human chondrocytes obtained from ATCC (CRL-2847, Manassas, VA) were cultured in DMEM (Gibco, Rockville, MD) supplemented with 10% FBS (Gibco, Rockville, MD), 1% penicillin and streptomycin (Gibco, Rockville, MD) at 37 °C with 5% CO_2_. At ~80% confluency, human chondrocytes cells (lower chamber of the Transwell unit) were co-cultured with hWJ-MSCs for 24 h in the SFM or SCM. hWJ-MSCs were seeded (1 × 10^5^/mL) into the upper chamber of 6-well transwell inserts (BD Falcon). After a 24-hr incubation period, cells were harvested through trypsinization (0.25%, Gibco-Invitrogen) and were washed with DPBS (Gibco, Rockville, MD).

### Cell proliferation assay

Cell proliferation was analyzed using Cell Counting Kit-8 (CCK-8, Dojindo Molecular Technologies, Kumamoto, Japan). Human chondrocyte cells (1 × 10^5^/mL, the lower chamber of the Transwell unit) co-cultured with hWJ-MSCs (1 × 10^5^/mL, the upper chamber of the Transwell unit) in 6-well-transwell for 24 h. Then 10% CCK-8 solution was added to each well, and cells were incubated for 4 h at 37 °C with 5% CO_2_. The reaction solution (100 μl each) was then transferred to a 96-well plate and was analyzed by measuring the absorbance at 450 nm using a microplate reader (Bio-Rad X-Mark spectrophotometer, Hercules, CA).

### Western blot analysis

Cell were lysed in RIPA buffer (Fisher Scientific, Pittsburgh, PA) in the present of Xpert protease inhibitor cocktail (GenDEPOT, Barker, TX). Proteins were separated by 4–20% Tris-Glycine gels (Bio-rad, Hercules, CA) and transferred to a nitrocellulose membrane. The antibodies tested included the anti-cyclin D1 antibody, anti-p53 antibody, (Cell Signaling, Danvers, MA), anti-SOX9 antibody (Santa Cruz Biotechnology, Dallas, TX), anti-MMP3 antibody, anti-MMP13 antibody (Abcam, Cambridge, MA) and β-actin antibody (Sigma-Aldrich, St Louis, MO) at 4 °C overnight. After washing, the membranes were incubated with a secondary antibody (goat anti-mouse IgG-HRP; goat anti-rabbit IgG-HRP; Sigma-Aldrich, St Louis, MO) for 1 h at RT. Blots were developed using ECL (Thermo Scientific Pierce, Rockford, IL) and protein bands were obtained by exposure to LAS-4000 image detection system (Fujifilm, Tokyo, Japan).

### Bioinformatic analysis

The identified proteins were analyzed using ProteinCenter bioinformatic tools (Proxeon Bioinformatics, http://www.cbs.dtu.dk/services). We made several protein sequences in one FASTA format file and submitted it to each program. SignalP (version 4.0, http://www.cbs.dtu.dk/services/SignalP4.0) was used to predict the presence of signal peptides in the identified proteins (D-cut-off values for SignalP-noTM networks >0.45 or SignalP-TM networks >0.5 as the default cut-off for signal peptide = ‘Yes’)^51^. The SecretomeP program (version 2.0, http://www.cbs.dtu.dk/services/SecretomeP2.0) was used to predict the possibility of nonclassical protein secretion (SignalP signal peptide = ‘No; and SecretomeP score >0.6 in mammal proteins)^52^. In addition, the TMHMM program (version 2.0, http://www.cbs.dtu.dk/services/TMHMM2.0) was used to predict transmembrane helices in integral membrane proteins^53^. The exosome proteins were defined as the proteins published at least 100 papers in EVpedia (http://evpedia.info)^17^. To predict the subcellular localization of identified proteins, CELLO (version 2.5, http://cello.life.nctu.edu.tw/) was used^54^.

Ingenuity Pathway Analysis (IPA, www.Ingenuity.com/) was used to carry out upstream regulator analysis of the hWJ-MSC secretome data. Uploaded data for upstream regulator analysis contains UniProtKB accession and the log2 ratio of identified proteins. Predicted upstream regulators with a Z-score above 2 and a p-value of overlap below 0.01 were considered significantly activated.

## Supplementary information

Supplementary Inforamtion

Supplementary Table S1

Supplementary Table S2
