# Supplementary material for: Comparative analysis of differentially secreted proteins in serum-free and serum-containing media by using BONCAT and pulsed SILAC
Source: Sci Rep. 2019 Feb 28;9:3096. doi: 10.1038/s41598-019-39650-z (PMC6395664; doi:10.1038/s41598-019-39650-z)
Supplement: Supplementary file 1 — Supplementary Inforamtion [file 41598_2019_39650_MOESM1_ESM.pdf]

Supporting Information

Comparative analysis of differentially secreted proteins in serum-free and serum-containing media by using BONCAT and pulsed SILAC

Jihye Shin[a,1], Jiheon Rhim[b,c], Yumi Kwon[a,d], Sun Young Choi[b,c,e], Sungho Shin[a,f], Chul-Won Ha[b,c,e] and Cheolju Lee[a,f,g*]

[a] Center for Theragnosis, Korea Institute of Science and Technology, Seoul 02792, Korea
[b] Department of Orthopedic Surgery, Samsung Medical Center, Sungkyunkwan University School of Medicine, Seoul 06351, Korea
[c] Stem Cell & Regenerative Medicine Institute, Samsung Medical Center, Seoul 06351, Korea
[d] Department of Life Science and Research Institute for Natural Sciences, Hanyang University, Seoul 04763, Korea
[e] Department of Health Sciences and Technology, SAIHST, Sungkyunkwan University, Seoul 06351, Korea
[f] Department of Converging Science and Technology, KHU-KIST, Kyung Hee University, Seoul 02447, Korea
[g] Division of Bio-Medical Science & Technology, KIST School, Korea University of Science and Technology, Seoul 02792, Korea

[1] Current address: Advanced Medical Research Center, Yokohama City University, Fukuura 3-9, 8 Kanazawa, Yokohama 236-0004, Japan

[*] **Corresponding author**

Tables of contents

Figure S1. AHA labeling and cell viability ········ 2
Figure S2. Optimization of CuAAC enrichment of secreted proteins ········ 3
Figure S3. Peptide sequence matches (PSM) of MS data for the secretome of BONCAT-pSILAC ········ 4
Figure S4. Proteins identified in three replicate experiments of U87MG and hWJ-MSC secretomes ········ 5
Figure S5. The percentage of FBS contamination inferred by PSMs matched to bovine proteins ········ 6
Figure S6. Growth of U87MG and hWJ-MSC as measured by cell counting ········ 7
Figure S7. Full-length western blot images of Fig. 3d and Fig. 4f ········ 8

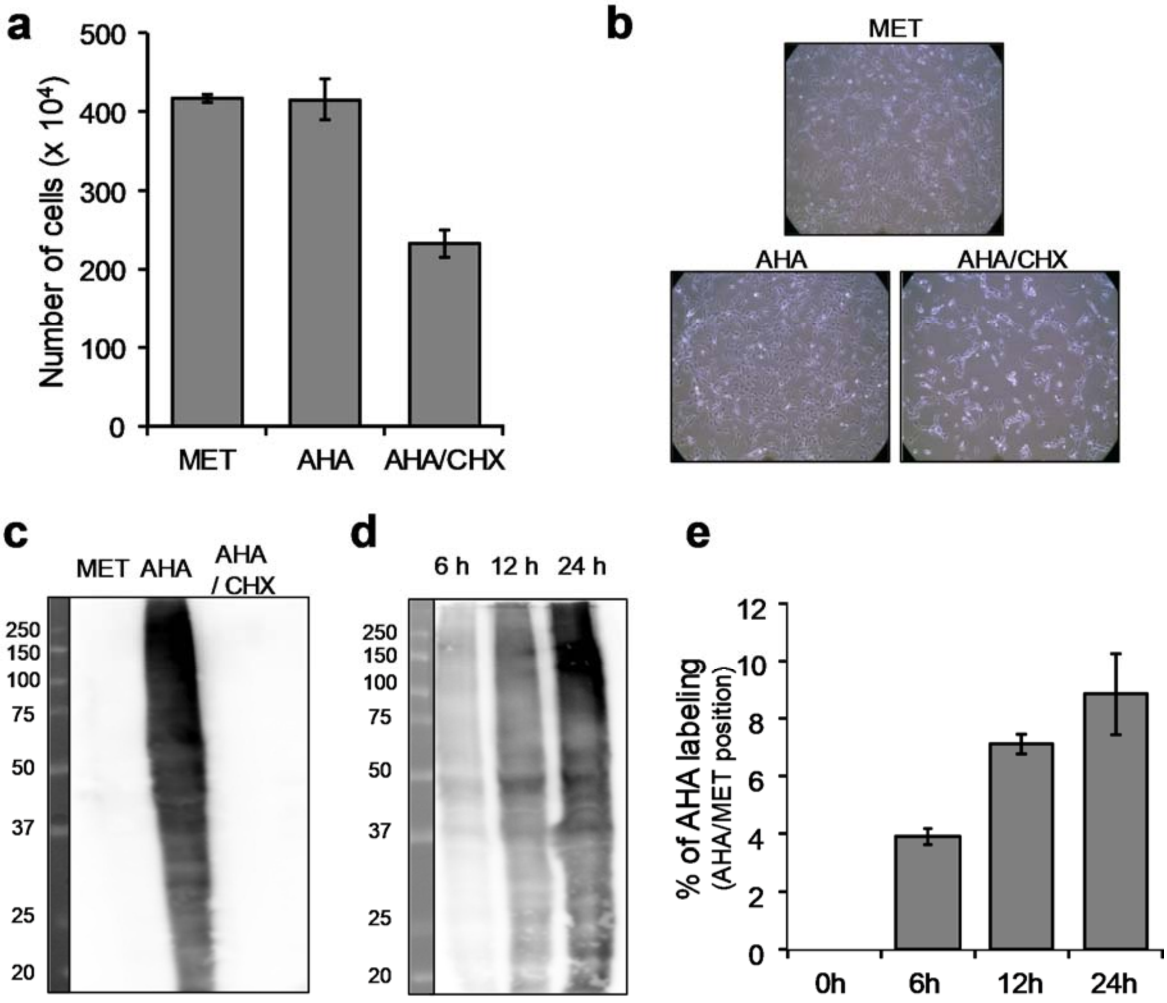

Figure S1. AHA labeling and cell viability. (a-c) U87MG cell lysates incubated for 24 h in media with 10% FBS and either 1 mM methionine (MET), 1 mM AHA (AHA) or 1 mM AHA and 10 μg/mL cycloheximide (AHA/CHX). The number **(a)** and morphologies **(b)** of U87MG cells were similar between AHA and methionine-treated cells. **(c)** CuAAC was performed on U87MG cell lysates, and the lysate was analyzed by western blot using an anti-biotin antibody. **(d-e)** AHA incorporation rate according to cell culture time as determined by western blot (d) and LC-MS/MS (e).

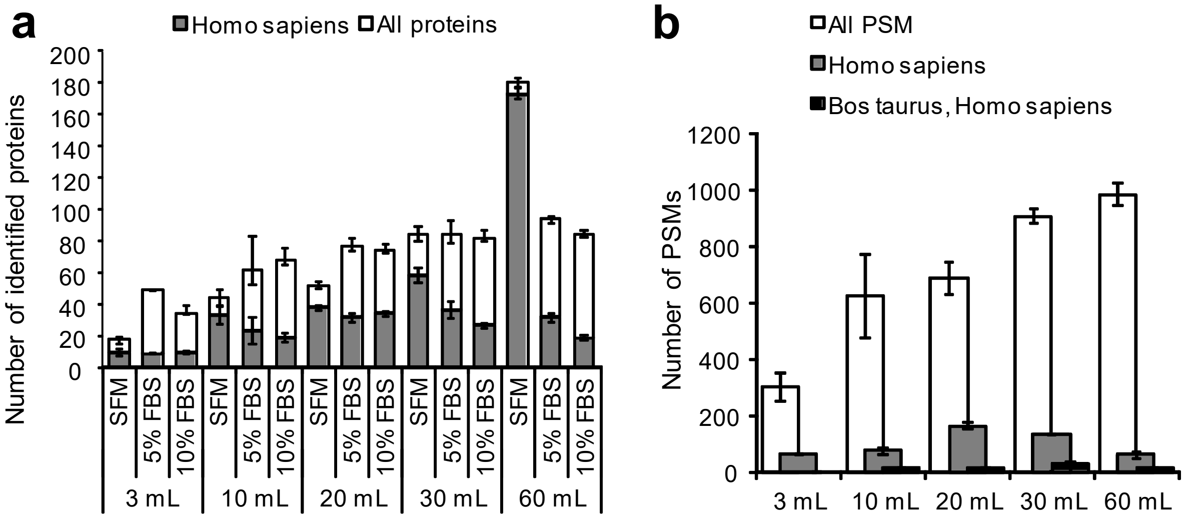

Figure S2. Optimization of CuAAC enrichment of secreted proteins. (a) Shown are the number of identified proteins after CuAAC enrichment of the conditioned media concentrated from 3, 10, 20, 30 and 60 mL at three different conditions (SFM, 5% FBS-containing medium, 10% FBS-containing medium). **(b)** Number of PSMs identified at 10% FBS-containing medium condition. Grey bar: PSMs matched to human sequence. Black bar: PSMs matched to homologous sequences between human and bovine. Note that a Human-FBS composite database was used for the analysis of MS data.

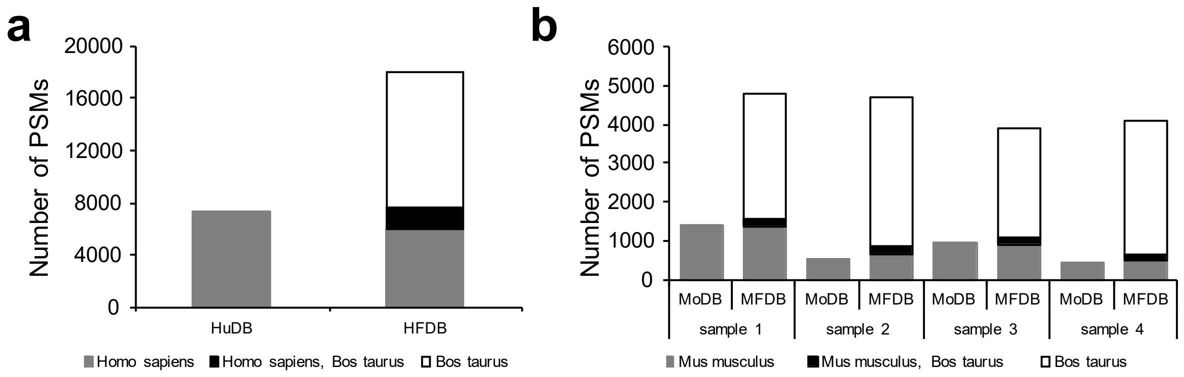

Figure S3. Peptide sequence matches (PSM) of MS data for the secretome of BONCAT-pSILAC. **(a)** The search results of U87MG secretome against human database (HuDB) and human-FBS database (HFDB) **(b)** Re-analysis results of a previous study (Eichelbaum, K.; Krijgsveld, J., Rapid temporal dynamics of transcription, protein synthesis, and secretion during macrophage activation. *Mol Cell Proteomics* **2014,** 13: 792-810) in which newly secreted proteins in 10% FBS-containing medium after mouse macrophage activation were analyzed. The MS data were searched against mouse Uniprot database (MoDB) and mouse-FBS database (MFDB). The FBS database contains 199 bovine protein entries. Black areas in both bar charts represent PSMs matching to both species (human and bovine in **a**; mouse and bovine in **b**) due to sequence homology.

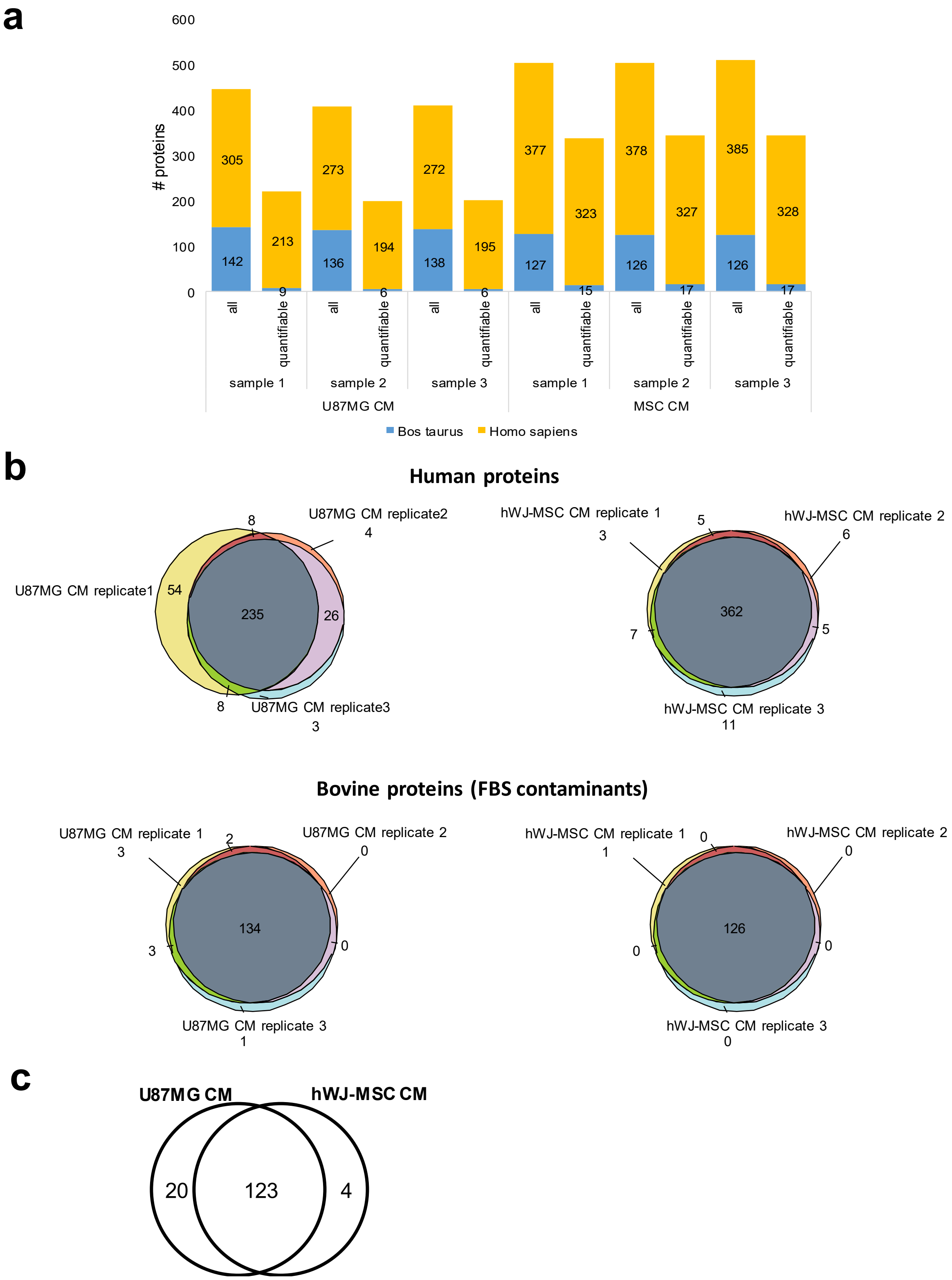

Figure S4. Proteins identified in three replicate experiments of U87MG and hWJ-MSC secretomes. (a) Bar graphs and **(b)** Venn diagram of identified human and bovine proteins. **(c)** Venn diagram of FBS contaminant proteins between the secretomes of two cells.

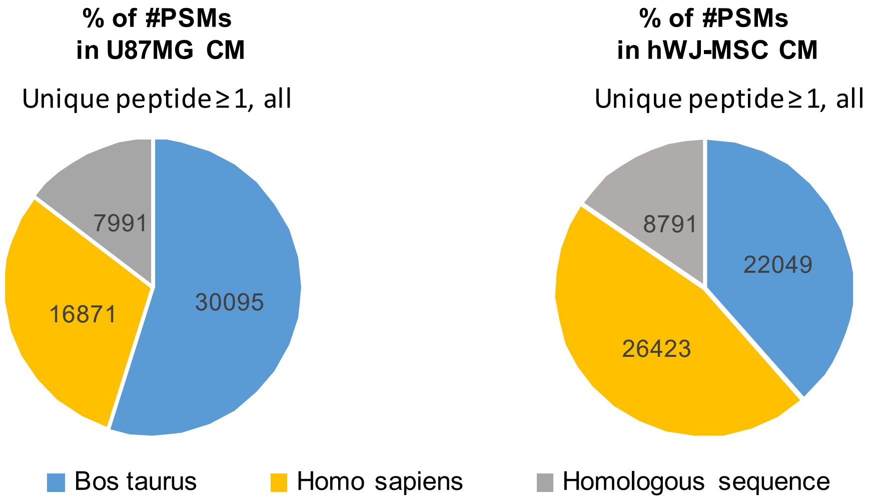

Figure S5. The percentage of FBS contamination inferred by PSMs matched to bovine proteis in U87MG and hWJ-MSC secretomes. Note that ‘homologous sequences’ are the peptides having identical sequences in both species. Therefore, it is impossible to tell at the peptide level where they are originated.

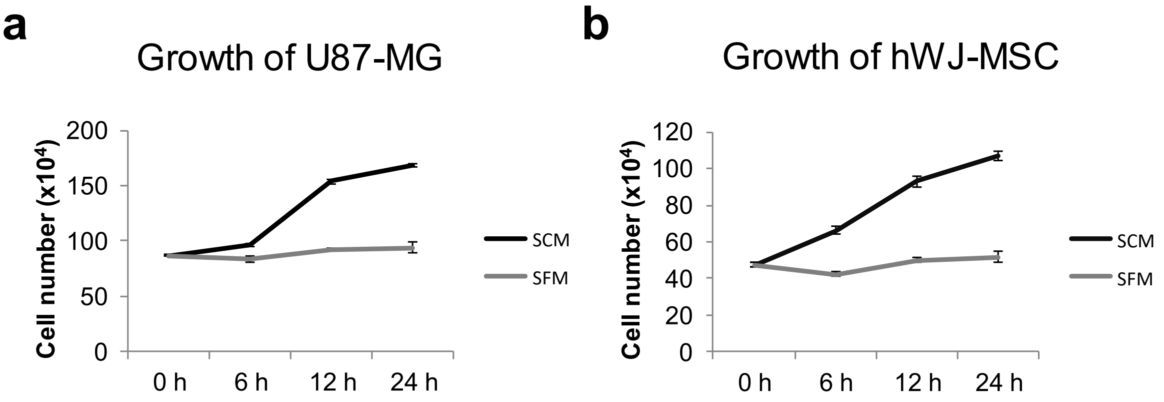

Figure S6. Growth of U87MG (a) and hWJ-MSC (b) as measured by cell counting. The relative area under the cell growth line from 0 h to 24 h were used as the normalization factors.

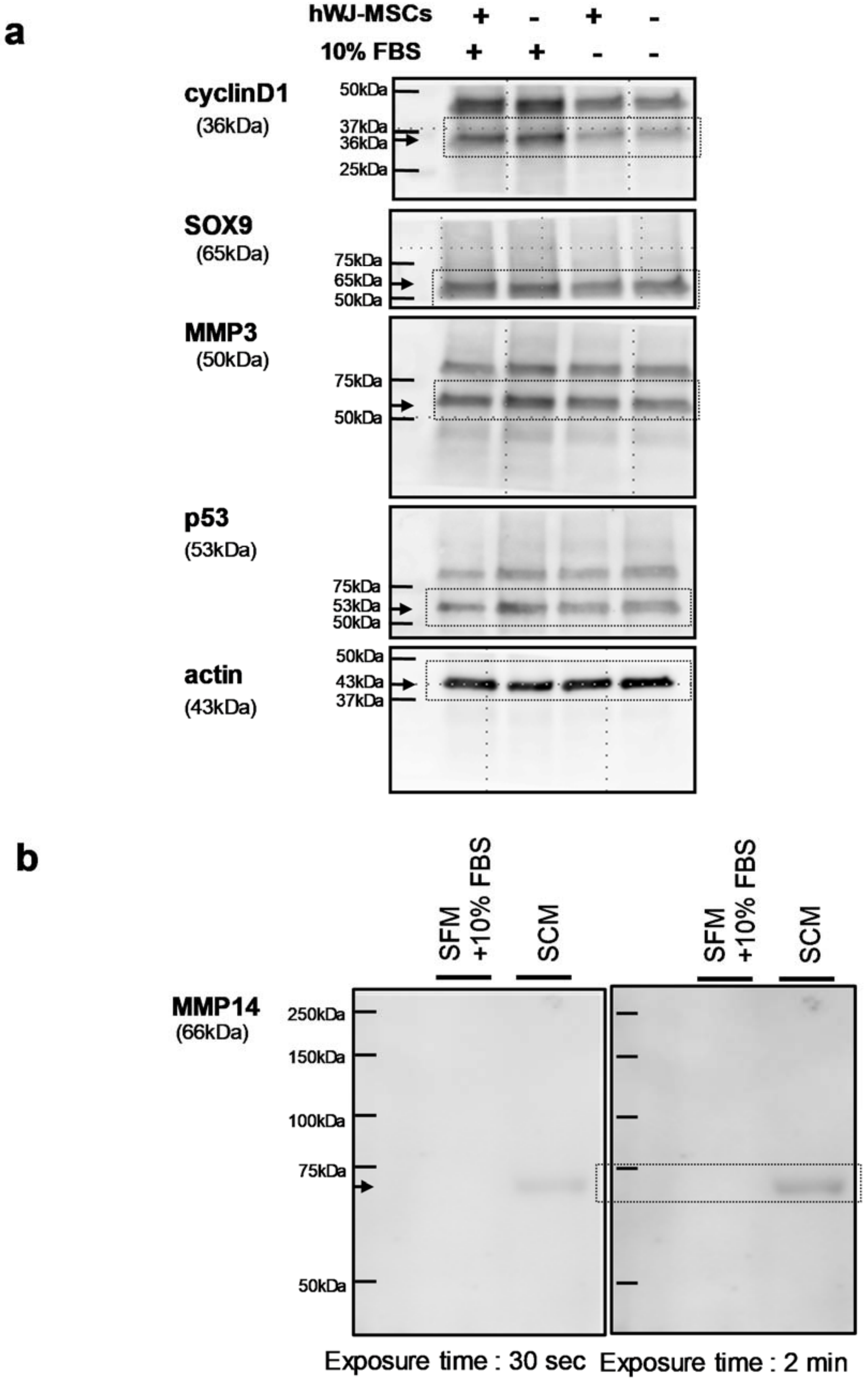

Figure S7. Full-length western blot images of Fig. 3d (a) and Fig. 4f (b).
